# Whitening the Hands and Face

**Published:** 1891-08

**Authors:** 


### WHITENING THE HANDS AND FACE.

The color of the skin is ruled by at least three influences, which are only in a very indirect manner under our own control. The cutaneous circulation is, perhaps, the most controllable ; if this be slow and impeded, the inner skin shows red or purplish through the outer skin ; whereas, a brisk and free blood current imparts that pinkish, living look which one calls flesh color. Hard and clean work with the hands and arms—combined, of course, with general health—is calculated to do all that is possible in this direction ; and making beds and applying "elbow grease" to mahogany furniture are two forms of work which are accounted especially efficacious in whitening the hands. Thickness of the outer skin is a second element conducive to whiteness. If people are naturally thin skinned, it is hard to see how they can produce any thing but an extremely limited local thickening. Some of the numerous emollient skin lotions may both stimulate the blood current and retard the desquamation which is perpetually going on, but I should expect nothing permanent from these applications. A third requisite for whiteness is absence of natural pigment. When a method of removing it has been discovered, a great many Ethiopians will be changing their skins. Meanwhile, we shall have to endure the presence of such pigment—black, brown, yellow, red or copper—as nature has placed under our skin. A certain pallor can generally be produced by gloves, idleness, indoor life, parasols and closed carriages; moreover, the blue veins on the lardy, or let us say waxy, white, have a certain look of delicacy and refinement. There is another method of securing paleness, though it cannot be trusted not to run to yellow. It is the paleness of ill health and anæmia; and semi-starvation on foods which are both innutritious and indigestible is the plan for securing it; at least, the most pallid girl I ever saw—one who looked as if she were modelled in spermaceti ointment,—was reported to have reduced herself to that condition by a sustained diet of dry, hard rice grains and cold water, taken with a view of producing a pale, pearly complexion.

